# Supplementary material for: Prospective multicenter study using artificial intelligence to improve dermoscopic melanoma diagnosis in patient care
Source: Commun Med (Lond). 2024 Sep 11;4:177. doi: 10.1038/s43856-024-00598-5 (PMC11387610; doi:10.1038/s43856-024-00598-5)
Supplement: Supplementary file 2 — Supplementary Information [file 43856_2024_598_MOESM2_ESM.pdf]

Supplementary Information

Supplementary Methods

Dataset description

The four hardware settings across the clinics were as follows:

- Setup1: HEINE IC1 dermatoscope with an Apple iPhone7
- Setup2: HEINE DELTAone dermatoscope with an Apple iPhone SE
- Setup3: HEINE Delta30 dermatoscope with an Apple iPhone 7
- Setup4: HEINE DELTAone dermatoscope with an Apple iPhone8

The original images were automatically cropped to exclude large parts of the black image margin which originates from dermoscopy and subsequently resized to 512x512, 768x768, and 1024x1024 pixels respectively for model inference.

Since the original location annotations are more fine-grained than the required input for the models requiring metadata, the following mapping is used (location grouped: location1, location2, …):

- anterior torso: abdomen, chest
- head/neck: face, scalp, neck
- lower extremity: leg (knee and below), thigh, foot
- oral/genital: genitalia
- palms/soles: palms, soles
- posterior torso: back, buttock
- upper extremity: arm, forearm, hand

Supplementary Table 1. Histopathologically verified diagnosis across the technical domain (i.e. camera setup) and data source (i.e. hospital).

Technical Domain (TD)	Data Source	Non-melanoma	Melanoma
TD1	Hospital 1	322	245
	Hospital 2	210	55
	Hospital 3	27	39
	**Combined**	**559**	**339**
TD2	Hospital 4	92	123
	Hospital 5	75	31
	**Combined**	**167**	**154**
TD3	Hospital 6	109	67
	Hospital 7	83	93
	**Combined**	**192**	**160**
TD4	Hospital 8	242	97
	**Combined**	**242**	**97**

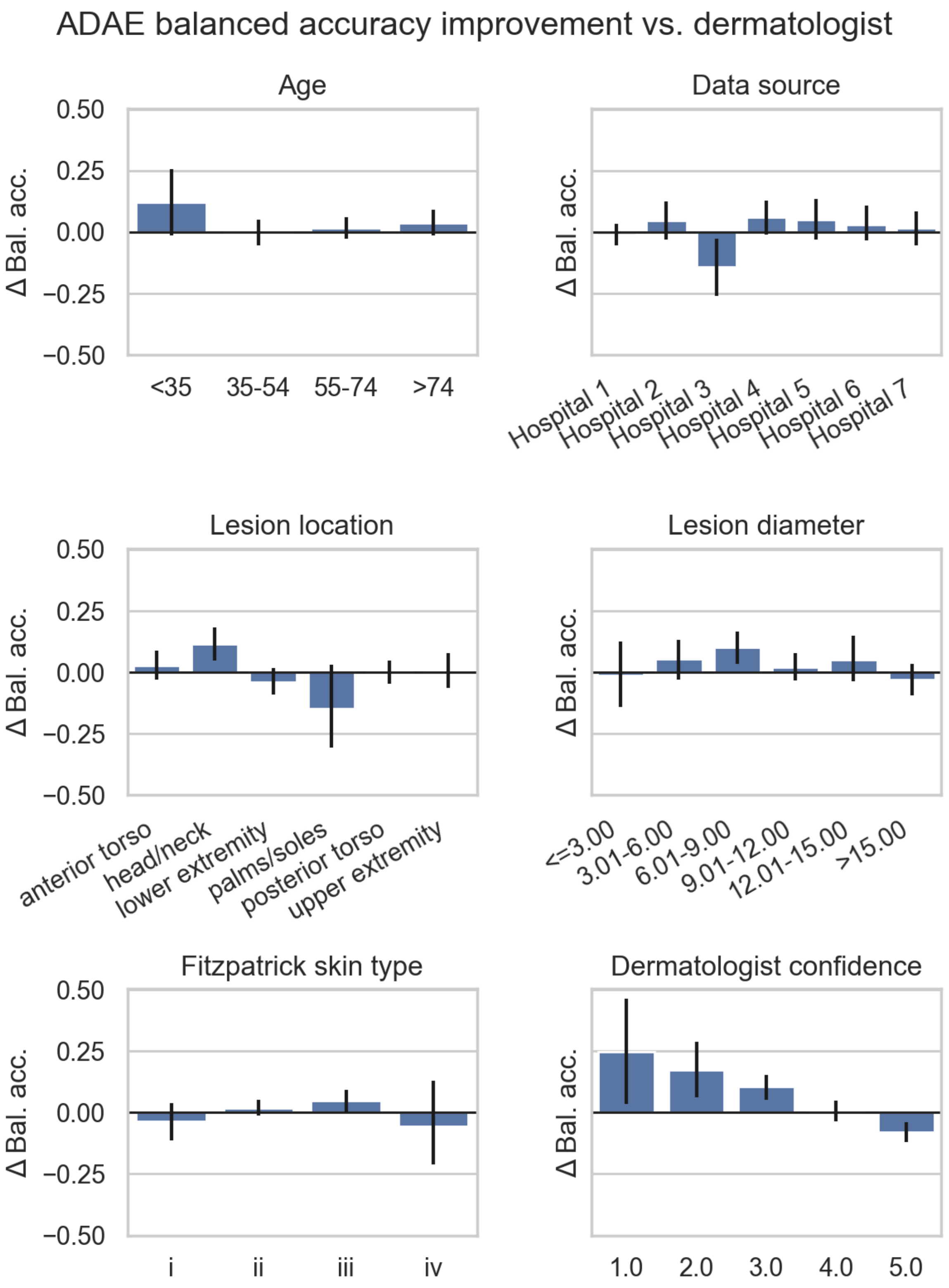

Supplementary Fig. 1. ADAE balanced accuracy improvements, stratified by patient age (top left), clinic (top right), lesion location (center left), lesion diameter (center right), patient Fitzpatrick skin type (bottom left), and dermatologist confidence (bottom right). Error bars indicate the 95% confidence intervals (CI).

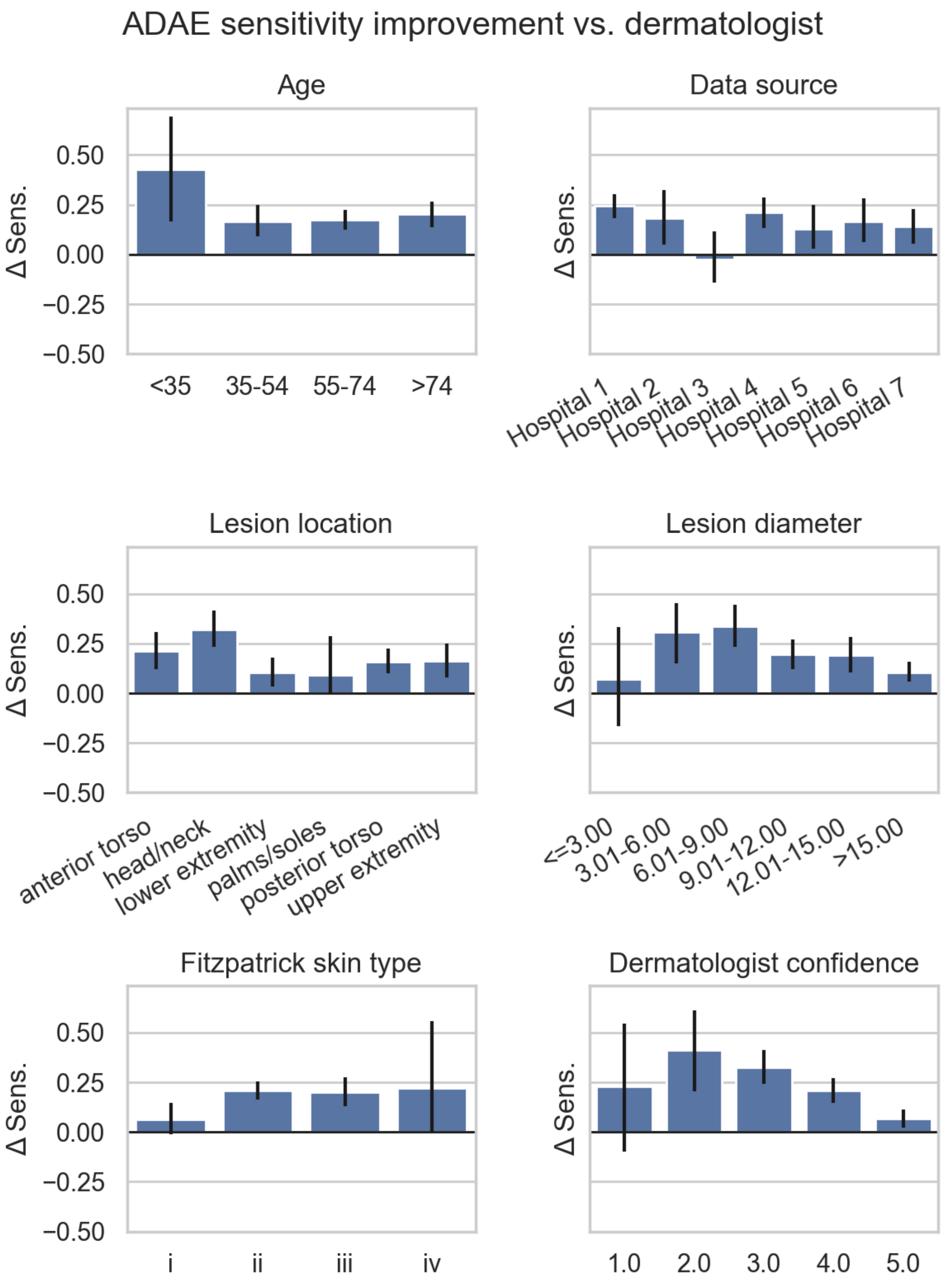

Supplementary Fig. 2. ADAE sensitivity improvements, stratified by patient age (top left), clinic (top right), lesion location (center left), lesion diameter (center right), patient Fitzpatrick skin type (bottom left), and dermatologist confidence (bottom right). Error bars indicate the 95% CIs.

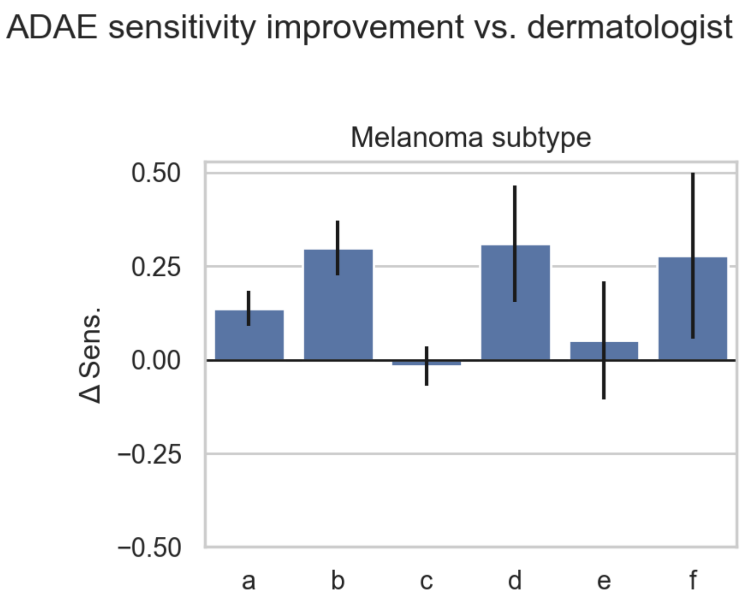

Supplementary Fig. 3. ADAE sensitivity improvements, stratified by Melanoma subtypes. Error bars indicate the 95% CIs. a: superficial spreading, b: others, c: nodular, d: lentigo maligna, e: acral lentiginous, f: combined forms

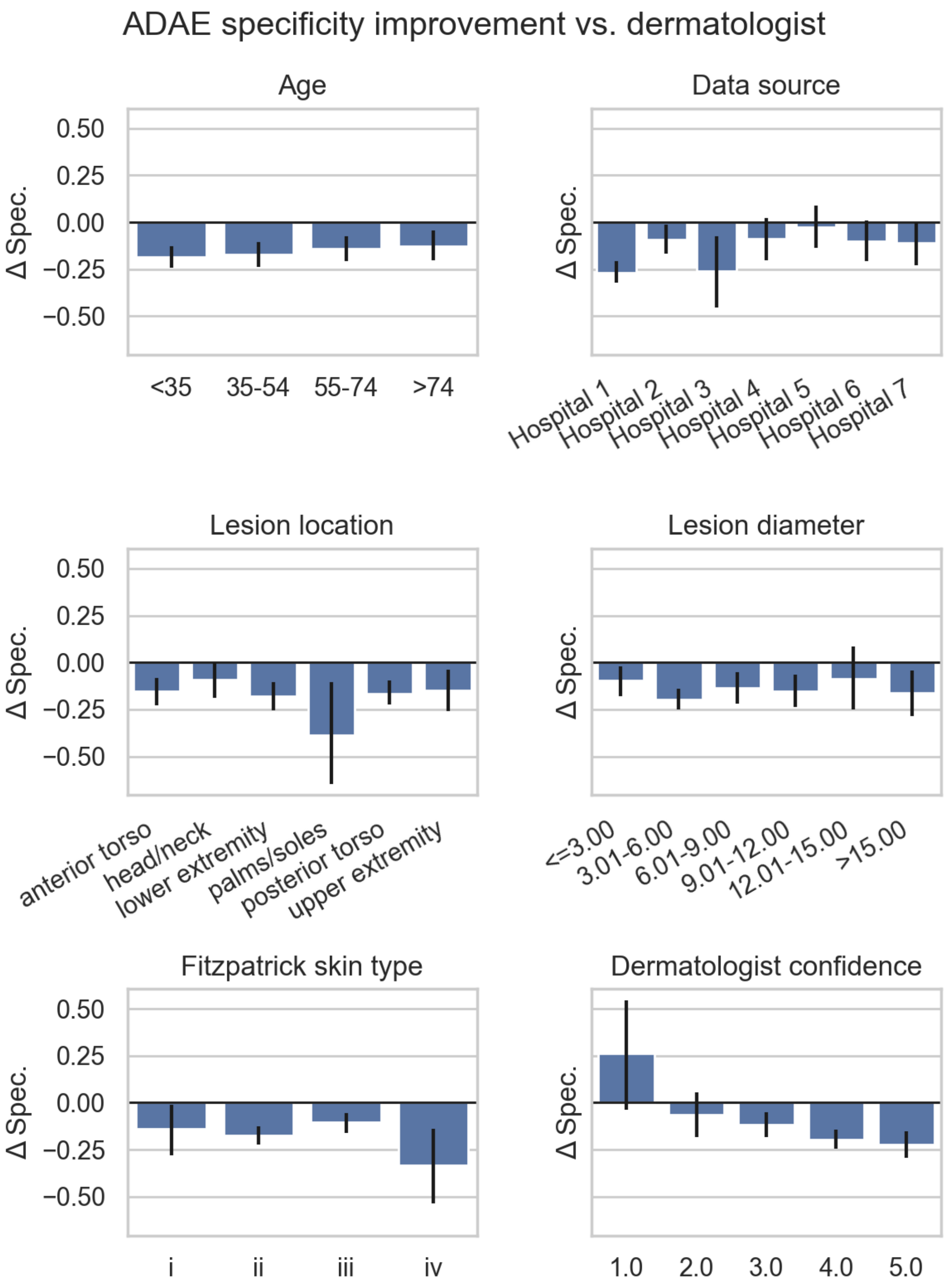

Supplementary Fig. 4. ADAE specificity improvements, stratified by patient age (top left), clinic (top right), lesion location (center left), lesion diameter (center right), patient Fitzpatrick skin type (bottom left), and dermatologist confidence (bottom right). Error bars indicate the 95% CIs.

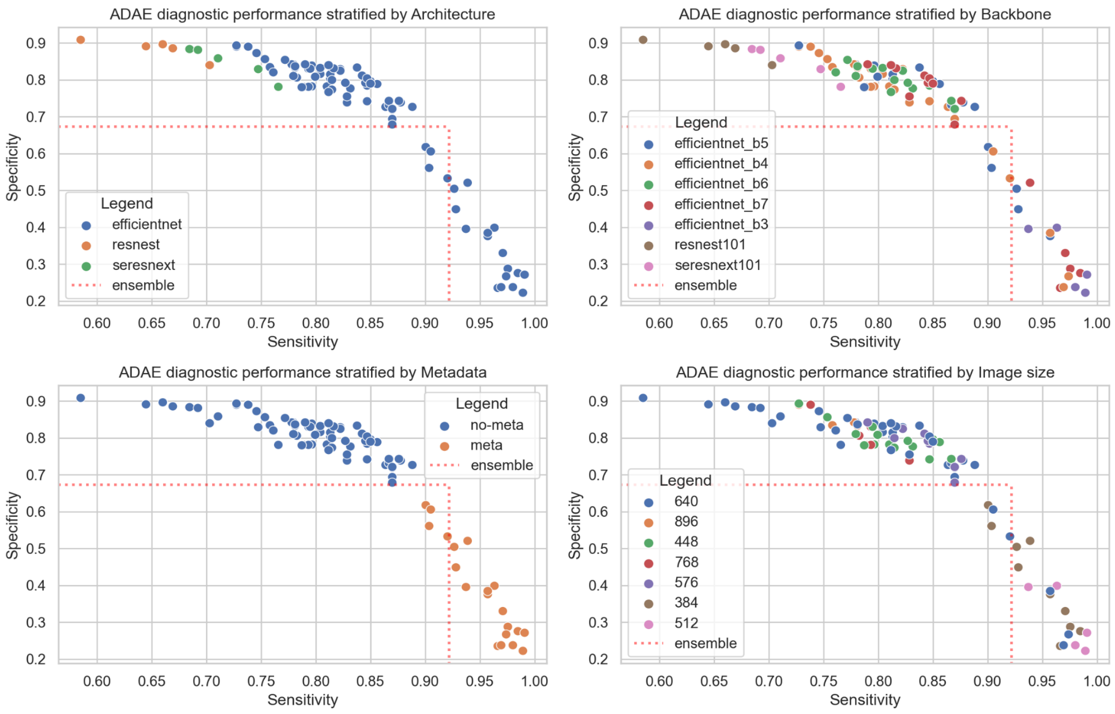

Supplementary Fig. 5. ADAE's diagnostic performance, stratified by architecture (top left), backbone (top right), metadata (bottom left), and image size (bottom right).

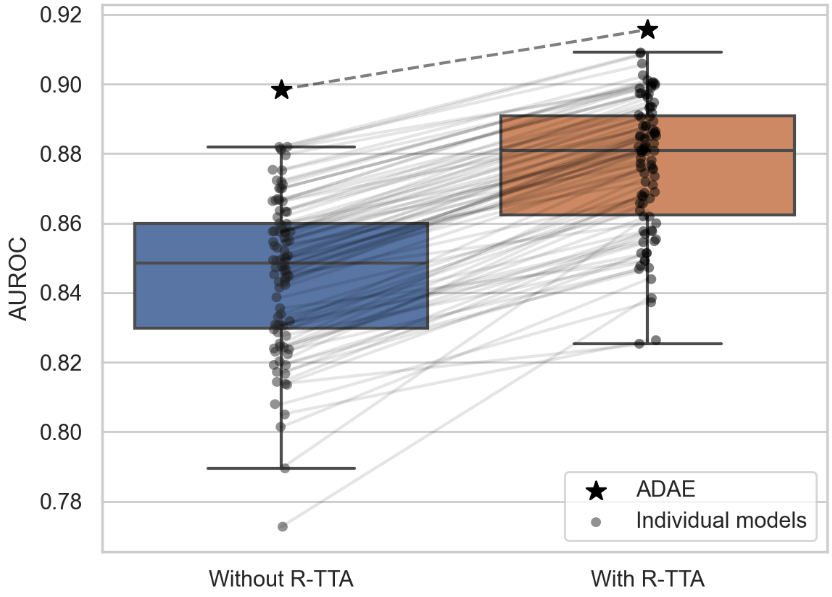

Supplementary Fig. 6. Impact of R-TTA: diagnostic accuracy of individual models. AUROC (diagnostic accuracy) of ADAE and its individual models with and without R-TTA. Each box extends from the lower to the upper quartile of the 1000 bootstrap iterations, with a line at the median. In addition, whiskers and fliers indicate the range and any outliers. R-TTA: real test-time augmentation, AUROC: area under the receiver operating characteristic curve.

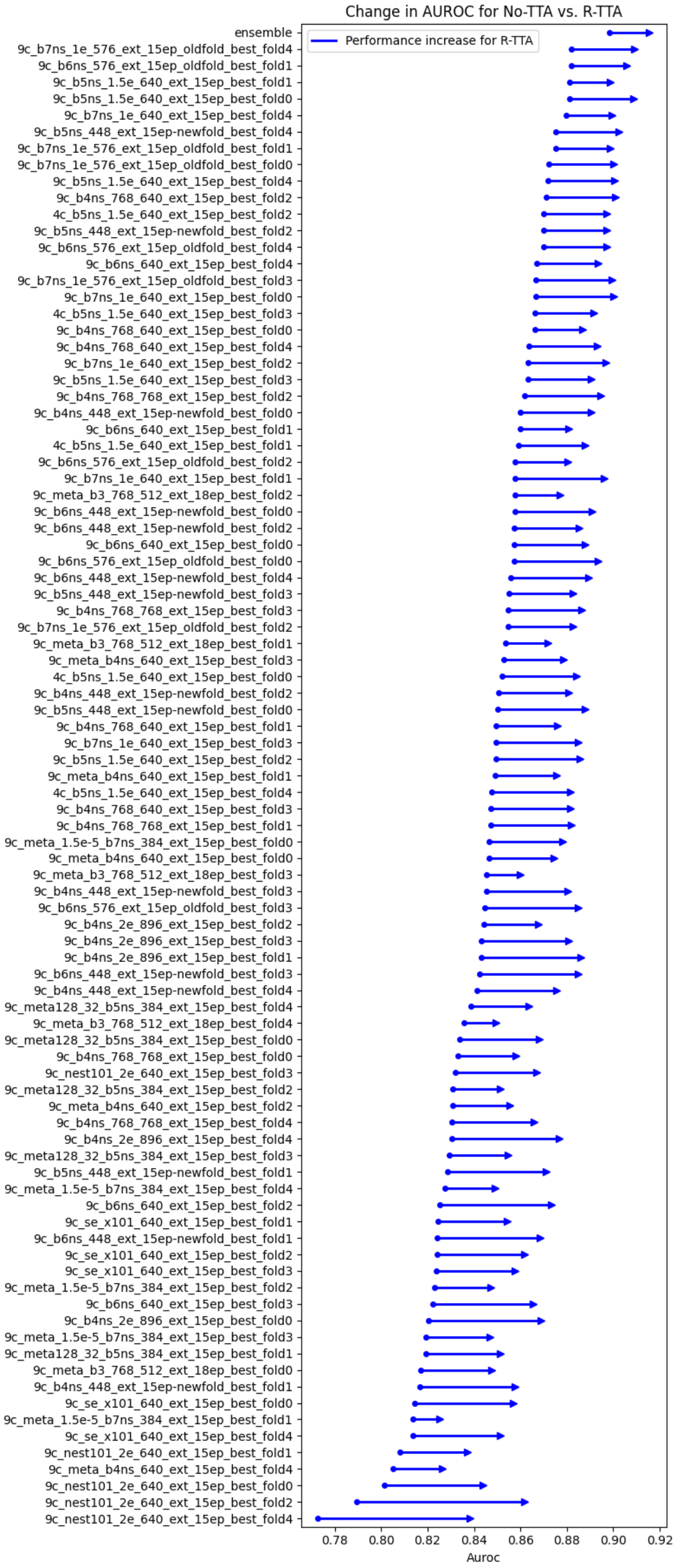

Supplementary Fig. 7. Impact of R-TTA: Change in diagnostic accuracy for individual models. The change in AUROC (diagnostic accuracy) for each individual model and the ensemble itself when utilizing R-TTA. R-TTA: real test-time augmentation, AUROC: area under the receiver operating characteristic curve.

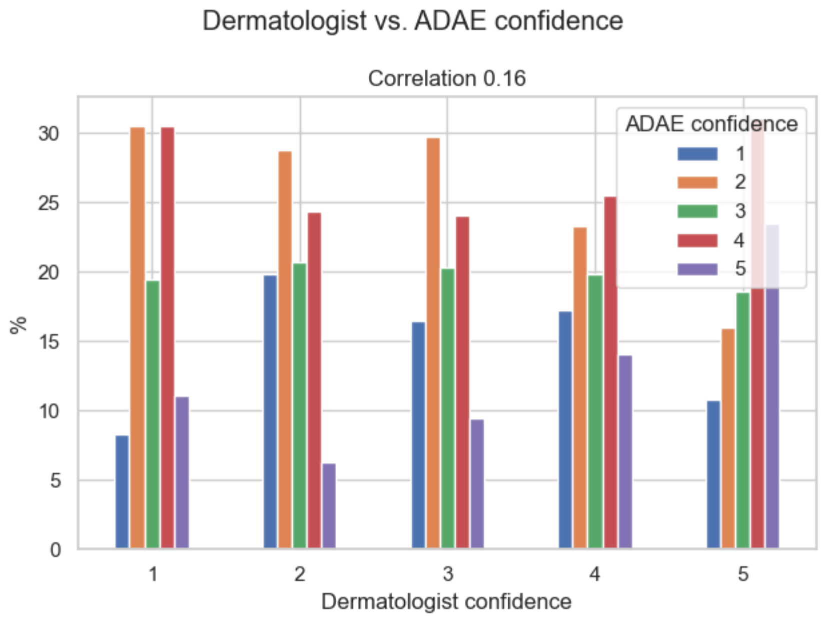

Supplementary Fig. 8: Relative distribution of Dermatologist vs. ADAE confidence scores.

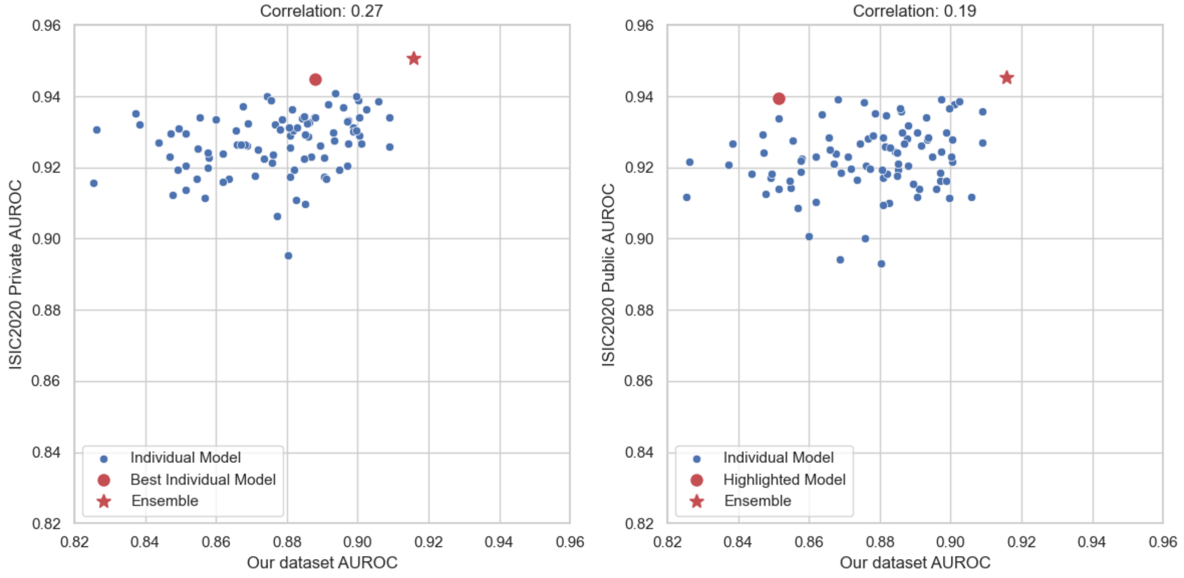

Supplementary Fig. 9: Correlation analysis. Correlation between the AUROC scores of the individual models of ADAE (without R-TTA) as well as the ensemble itself for ISIC 2020 test (left) and validation (right) set vs. our test set. AUROC: area under the receiver operating characteristic curve, R-TTA: real test-time augmentation
