# Supplementary material for: Prospective multicenter study using artificial intelligence to improve dermoscopic melanoma diagnosis in patient care
Source: Commun Med (Lond). 2024 Sep 11;4:177. doi: 10.1038/s43856-024-00598-5 (PMC11387610; doi:10.1038/s43856-024-00598-5)
Supplement: Supplementary file 3 — Description of Additional Supplementary Files [file 43856_2024_598_MOESM3_ESM.pdf]

Description of Additional Supplementary Files

File name- Supplementary Data 1

File description- The different technical setups (technical domain) are explained in detail in the Supplementary Methods

File name- Supplementary Data 2

File description- The numerical data underlying Figure 1 (source data) can be found in Supplementary Data 2.
