# Supplementary material for: Prospective multicenter study using artificial intelligence to improve dermoscopic melanoma diagnosis in patient care
Source: Commun Med (Lond). 2024 Sep 11;4:177. doi: 10.1038/s43856-024-00598-5 (PMC11387610; doi:10.1038/s43856-024-00598-5)
Supplement: Supplementary file 6 — Reporting Summary [file 43856_2024_598_MOESM6_ESM.pdf]

nature portfolio

Corresponding author(s): Titus J. Brinker

Last updated by author(s): Jun 19, 2024

Reporting Summary

Nature Portfolio wishes to improve the reproducibility of the work that we publish. This form provides structure for consistency and transparency in reporting. For further information on Nature Portfolio policies, see our Editorial Policies and the Editorial Policy Checklist.

Statistics

For all statistical analyses, confirm that the following items are present in the figure legend, table legend, main text, or Methods section.

n/a	Confirmed	
☐	☒	The exact sample size (*n*) for each experimental group/condition, given as a discrete number and unit of measurement
☐	☒	A statement on whether measurements were taken from distinct samples or whether the same sample was measured repeatedly
☐	☒	The statistical test(s) used AND whether they are one- or two-sided<br>*Only common tests should be described solely by name; describe more complex techniques in the Methods section.*
☒	☐	A description of all covariates tested
☒	☐	A description of any assumptions or corrections, such as tests of normality and adjustment for multiple comparisons
☐	☒	A full description of the statistical parameters including central tendency (e.g. means) or other basic estimates (e.g. regression coefficient) AND variation (e.g. standard deviation) or associated estimates of uncertainty (e.g. confidence intervals)
☐	☒	For null hypothesis testing, the test statistic (e.g. F, t, r) with confidence intervals, effect sizes, degrees of freedom and P value noted<br>*Give P values as exact values whenever suitable.*
☒	☐	For Bayesian analysis, information on the choice of priors and Markov chain Monte Carlo settings
☒	☐	For hierarchical and complex designs, identification of the appropriate level for tests and full reporting of outcomes
☒	☐	Estimates of effect sizes (e.g. Cohen's *d*, Pearson's *r*), indicating how they were calculated

Our web collection on statistics for biologists contains articles on many of the points above.

Software and code

Policy information about availability of computer code

Data collection	No software was used to collect data.
Data analysis	Python 3.11.5, SciPy 1.11.2, R 4.3.1

For manuscripts utilizing custom algorithms or software that are central to the research but not yet described in published literature, software must be made available to editors and reviewers. We strongly encourage code deposition in a community repository (e.g. GitHub). See the Nature Portfolio guidelines for submitting code & software for further information.

Data

Policy information about availability of data

All manuscripts must include a data availability statement. This statement should provide the following information, where applicable:

- Accession codes, unique identifiers, or web links for publicly available datasets
- A description of any restrictions on data availability
- For clinical datasets or third party data, please ensure that the statement adheres to our policy

The pretrained ADAE algorithm is publicly available via GitHub https://github.com/ISIC-Research/ADAE. The SIIM-ISIC 2020 data is publicly available at https://challenge2020.isic-archive.com/. Our dataset is available from the corresponding author on reasonable request.

Human research participants

Policy information about studies involving human research participants and Sex and Gender in Research.

Reporting on sex and gender	Sex was considered in this study design. The study comprises 985 male, and 731 female participants.
Population characteristics	The patients' age at diagnosis ranged from 18 to 96 years, with a median age of 62 years. Of the 1716 participants, 1320 had no personal history of melanoma, while 335 did, and it is unknown for the remaining 61 participants. 1495 had no family history of melanoma, while 123 had, and it is unknown for the remaining 98 participants.
Recruitment	There was no explicit recruitment of participants. Only patients that went on their own initiative for a suspected diagnosis or a routine examination to the university hospitals were included in the study, and only if present with at least one melanoma suspicious lesion.
Ethics oversight	University Hospital Berlin, University Hospital Dresden, University Hospital Erlangen, University Hospital Essen, University Hospital Mannheim, University Hospital München, University Hospital Regensburg, University Hospital Würzburg

Note that full information on the approval of the study protocol must also be provided in the manuscript.

Field-specific reporting

Please select the one below that is the best fit for your research. If you are not sure, read the appropriate sections before making your selection.

☒ Life sciences ☐ Behavioural & social sciences ☐ Ecological, evolutionary & environmental sciences

For a reference copy of the document with all sections, see nature.com/documents/nr-reporting-summary-flat.pdf

Life sciences study design

All studies must disclose on these points even when the disclosure is negative.

Sample size	The study was prospective with n >= 200 per clinic, with 8 distinct clinics total. The final dataset consists of 1910 melanoma suspicious lesions.
Data exclusions	Patients were excluded if the melanoma-suspicious lesions had undergone pre-biopsy procedures, were located near the eye or beneath the fingernails or toenails, or had person-identifying features (such as tattoos) in the immediate vicinity of the lesion due to data privacy concerns.
Replication	n.a.
Randomization	n.a.
Blinding	n.a.

Reporting for specific materials, systems and methods

We require information from authors about some types of materials, experimental systems and methods used in many studies. Here, indicate whether each material, system or method listed is relevant to your study. If you are not sure if a list item applies to your research, read the appropriate section before selecting a response.

Materials & experimental systems

n/a	Involved in the study	
☒	☐	Antibodies
☒	☐	Eukaryotic cell lines
☒	☐	Palaeontology and archaeology
☒	☐	Animals and other organisms
☒	☐	Clinical data
☒	☐	Dual use research of concern

Methods

n/a	Involved in the study	
☒	☐	ChIP-seq
☒	☐	Flow cytometry
☒	☐	MRI-based neuroimaging
